# Behavioural Factors As Predictors of Self-rated Health Among Polish Adolescent Girls

**DOI:** 10.34763/devperiodmed.20192302.109116

**Published:** 2019-07-08

**Authors:** Maria Jodkowska, Anna Oblacińska, Anna Dzielska, Hanna Nałęcz, Anna Fijałkowska

**Affiliations:** 1 Department of Child and Adolescent Health, Institute of Mother and Child, Warsaw, Poland; 2 Department of Cardiology, Institute of Mother and Child, Warsaw, Poland

**Keywords:** self-rated health, health behaviours, girls, zdrowie subiektywne, zachowania zdrowotne, dziewczęta

## Abstract

**Introduction:**

Self-rated health (SRH), an indicator which is extensively used in population studies, constitutes a measure of health closely linked to morbidity, mortality and overall health status and enjoys popularity in surveys monitoring adolescents. Most studies show that at puberty girls assess their health as worse than boys do, and the difference widens with age. Moreover, puberty is a crucial period for health, since it is the time when health risk behaviours are often initiated or become established.

**Aim:**

To analyse the associations between high scores on self-rated health among 15-year-old girls, participants of the Healthy Me programme, and their selected health behaviours.

**Material and methods:**

The study covered a group of 1173 second-grade female students from 48 lower secondary schools located in rural and urban areas of 16 voivodeships all over Poland. The participants answered questions about chronic diseases or disability, self-rated health, diet, leisure activities, physical activity and health risk behaviours. In the statistical analysis, the association between self-rated health and individual indicators of health behaviour was examined using logistic regression.

**Results:**

Two thirds of the girls assessed their health as excellent or good. Only approximately 5% of the respondents made the "extreme negative" assessment. In the final multivariate analysis, five factors remained important predictors of high self-rated health scores: regular participation in physical education classes, vigorous physical activity, daily breakfast consumption, consumption of fruit at least once a day and sleep for at least 8 hours a day.

**Conclusions:**

Regularparticipation in physical education classes, vigorousphysical activity, consumption of breakfast and fruit every day, as well as sleep for at least 8 hours a day are powerful predictors of high scores on self-rated health of 15-year-old adolescent girls.

Public health activities aimed at adolescents should focus on the positive aspects of health and a lifestyle paying special attention on pro health behaviours.

## Streszczenie

**Wstęp:**

Samoocena zdrowia, szeroko stosowana w badaniach populacyjnych, jest miernikiem zdrowia ściśle związanym z zachorowalnością, umieralnością i ogólnym stanem zdrowia. W badaniach ankietowych młodzieży często używany jest wskaźnik samooceny zdrowia SRH (self-rated health). Z większości badań wynika, że dziewczęta w okresie dojrzewania gorzej oceniają swoje zdrowie niż chłopcy, a różnica ta pogłębia się z wiekiem. Okres dojrzewania jest także decydującym okresem dla kształtowania się stanu zdrowia, ponieważ zachowania ryzykowne często są w tym czasie inicjowane lub utrwalane.

**Cel:**

Analiza związku między wysoką samooceną zdrowia 15-letnich dziewcząt, uczestniczek programu Zdrowa Ja, a wybranymi zachowaniami zdrowotnymi.

**Materiał i metoda:**

Badaniem objęto grupę 1 1173 uczennic II klas gimnazjum z 48 szkół zlokalizowanych na terenach miejskich i wiejskich w 16 województwach w Polsce. Uczestniczki odpowiadały na pytania dotyczące występowania u nich chorób przewlekłych lub niepełnosprawności, samooceny zdrowia oraz sposobu żywienia, spędzania wolnego czasu, aktywności fizycznej i zachowań ryzykownymi dla zdrowia. W analizie statystycznej, związek między samooceną zdrowia, a poszczególnymi wskaźnikami zachowań zdrowotnych zbadano za pomocą regresji logistycznej.

**Wyniki:**

Dwie trzecie dziewcząt oceniło swoje zdrowie jako doskonałe i dobre. Krańcowej, negatywnej oceny dokonało jedynie około 5% respondentek. W końcowym modelu wielowymiarowej regresji logistycznej jako istotne predyktory wysokiej samooceny zdrowia pozostały następujące czynniki: regularny udział w lekcjach wychowania fizycznego, intensywne ćwiczenia fizyczne, codzienne spożywanie śniadań, spożywanie owoców co najmniej raz dziennie oraz czas snu co najmniej 8 godzin na dobę.

**Wnioski:**

Regularne uczestnictwo w lekcjach WF, intensywna aktywnośćfizyczna, codzienne spożywanie śniadań i owoców oraz co najmniej 8 godzin snu są silnymi predyktorami wysokiej samooceny zdrowia 15-letnich dziewcząt. Działania na rzecz zdrowia publicznego skierowane do młodzieży powinny koncentrować się na pozytywnych aspektach zdrowia i życia, zwracając szczególną uwagę na zachowania prozdrowotne.

## Introduction

The self-rated health (SRH) indicator is extensively used in population studies. It is a popular measure of health, closely linked to morbidity, mortality and overall health status [1], and refers to a subjective perception of one’s health. The responses of adolescents to self-assessed questions about health seem to reflect their well-being, health behaviour or functional ability to use health services relatively well [2,3]. Subjective assessment of health seems to remain comparatively constant for several years, and thus may be used for predicting both the health of an individual in subsequent years as well as the occurrence of various risk factors in later life [4].

Puberty is a critical period for health, since health risk behaviours are often initiated or established at the time [5,6]. Questionnaire surveys of adolescents frequently use the SRH indicator. Although puberty is a rather “healthy” period of life, it is also the time when behaviours that may have a long-term influence on health and well-being in adulthood are formed. Although adolescents assess their health significantly better than adults, signalling any health problems in the second decade of life forecasts a worse general functioning in subsequent years, higher susceptibility to disease and a lower quality of life [7].

Most studies show that adolescent girls rate their health as worse than boys do, and this difference widens with age, remaining significant for young adults [3,8,9]. In a Polish 2018 study, one in four 15-year-old girls rate their health as worse than good [10].

The identification of relations between health behaviours and self-rated health in the population of adolescents is important for preparing appropriate interventions, aimed at changing the current lifestyle and sustaining it during adulthood.

## Aim

The aim of the study was to analyse the association between the high assessment of self-rated health among 15-year-old girls and their selected health behaviours.

## Material and methods

### Material

A survey was conducted of 1173 second-grade female students from 48 lower secondary schools in 16 voivodeships – in large cities (population over 100,000), smaller towns and rural areas in Poland. The stratified randomisation technique was used, controlled for domicile. Sampling ensured coverage of regions with different socioeconomic levels. It was the first of three surveys conducted using self-completion questionnaires administered in school classrooms in November 2017, to precede the launching of the *Healthy Me* intervention program.

The schools were randomly assigned to three groups: a null intervention group, partial intervention and full intervention group (12, 12 and 24 schools, respectively). 1198 girls were enrolled in the project:

– intervention group I (full intervention) – with an objective measurement of physical activity, receipt of feedback from a wearable device (a fitness band), dedicated mobile App (short messages about healthy lifestyle, gamification), and other activities (health education workshops at school) supporting physical activity and a healthy lifestyle – 24 schools (636 girls);
– intervention group II (partial intervention) – with objective measurement of physical activity, receipt of feedback from a wearable device (a fitness band), dedicated mobile App (short messages about healthy lifestyle), – 12 schools (277 girls);
– control group (null intervention) – objective measurement of physical activity, receipt of feedback from a wearable device (a fitness band) – 12 schools (285 girls).

The study obtained the approval of the Bioethical Committee of the Institute of Mother and Child1Opinion No 32/2017 of the Bioethical Committee at the Institute of Mother and Child of 22.06.2017 along with an Annex of 9.11.2017..

### Methods

The research tool was a questionnaire which was self-completed in the classroom. Participants answered the questions related to SRH, chronic diseases or disability confirmed by a doctor, as well as their diet, leisure time activities, sleep time, physical activity and risk behaviours.

Outcome variable – SRH was based on the question: *Would you say that your health is: excellent, good, fair, poor*. Then the answers: excellent and good were combined into the category „high self-rated health” while fair and poor into the category “low self-rated health”.

The question was adapted from the HBSC (Health Behaviour in School-aged Children) survey protocol. Its source is assumed to be the systematic review of studies published by Idler et al. [11].

The health behaviours of the girls were analysed in the study using questions about their diet, leisure time activity, physical activity and risk behaviours.

1. Consumption of breakfast, fruit and vegetables as well as family meals with parents:

*• How often do you usually have breakfast (more than a glass of milk, tea or other drink) on weekdays?* Response categories: I never have breakfast during the week; one day; two days;...; five days. The breakfast consumption frequency was analysed in the dichotomous split: girls having breakfast regularly (on five school days) and irregularly (less than 5 days).
- Two separate questions for fruit and vegetable consumption: *How many times a week do you usually eat fruits/vegetables?* Response categories: never; less than once a week; once a week; 2-4 days a week; 5-6 days a week; once a day, every day; every day, more than once. In subsequent analyses, a dichotomous split was applied: at least once a day (every day and more than once a day) and less than every day.
*• How often do you and your family usually have meals together?* Response categories: every day; most days; about once a week; less often; never. In subsequent analyses, a dichotomous split was applied: often (every day and on most days) and seldom (about once a week, less often, never).

2. Physical activity and sedentary behaviour (screen time)

- Physical activity was measured based on two indicators:

– MVPA (Moderate-to-Vigorous Physical Activity). The girls answered the following question: *Over the past 7 days, on how many days were you physically active for a total of at least 60 minutes per day?* Please add up all the time you spent doing physical activity each day. They marked their response on a visual scale with a range of from 0 to 7 days. MVPA was dichotomized into two categories: recommended PA level (7 days a week) and physical activity below recommendations.
– VPA (Vigorous Physical Activity) is an activity which results in substantial energy expenditure (< 6METs), out of breath and increased heart rate, e.g. running, fast cycling, fast swimming. The teenagers were asked the following question: *Outside school hours: how often do you usually exercise in your free time so much that you get out of breath or sweat? –There* were 7 response categories: every day; 4-6 times a week; 2-3 times a week; once a week; once a month; less than once a month; never. In subsequent analyses, the indicator was dichotomous: recommended VPA level 2-3 times a week or more, and below the recommendations (VPA< 2-3 times a week).

- Participation in physical education (PE) classes: The girls were asked: *In how many physical education classes did you participate in the previous school year?* with response categories: all or almost; more than half; half; less than half; almost none; none. Participation in PE classes was analysed in a dichotomous division: regularly (all and almost all classes), and irregularly (less often than in previous category).

Secondary schools in Poland are required to provide 3 PE classes (135 minutes) per week.

- Sedentary behaviour:

The girls were asked: *How many hours a day, do you usually spend in your free time*:

– *watching TV, videos (including YouTube or similar services), DVDs, and other entertainment on a screen?*
– *How many hours a day do you usually spend in your free time using electronic devices, such as computers, tablets (like iPad) or smartphones for other purposes, for example, homework, emailing tweeting Facebook, chatting surfing the internet?*

Each question concerned school days, with nine response categories from not at all to about 7 or more hours a day. In subsequent analyses, a dichotomous split was applied: less than two hours and over two hours. 3. Tobacco smoking, alcohol consumption and marijuana use.

The girls were asked: *on how many days (if any) they smoked cigarettes, drank alcohol, used marijuana in all their life?* response categories: never, 1-2 days, 3-5 days, 6-9 days, 10-19 days, 20-29 days, 30 days or more. In subsequent analyses, a dichotomous split was applied: never and with varied frequency.

4. Sleep

Sleep duration was determined based on two questions: *When do you usually go to bed if you have to go to school next morning?* (responses from not later than at 9.00 pm to 2.00 am and later) and: *When do you usually wake up on school mornings?* (responses from not later than 05.00 am to 08.00 am or later). According to experts [12] the recommended sleep amount for adolescents aged 13-18 years is 8-10 hours. In this study, a dichotomous split was applied: at least 8 hours and less than 8 hours.

### Controlled variable

The variable “chronic disease” was added as the controlled variable to the above-mentioned independent variables based on responses to the question: *Do you have any chronic (long-term) illnesses, are you handicapped or do you have any other health problems (e.g. diabetes, arthritis, serious allergy, cerebral palsy) which have been diagnosed by a doctor?-*, response categories – yes, no.

### Statistical analysis of data

The above-mentioned health behaviours were analysed in the groups of girls with high and low SRH scores, using cross tabulation. The differences between the groups were determined with the Chi2 test. The influence of 13 indicators of health behaviour (independent variables categorized into 2 independent dichotomized variables, where „1” meaning a positive level; each time the positive variable was chosen) on high SRH scores (dependent variable) was the analysed using univariate logistic regression analysis. The results of univariate analysis were presented as crude odds ratios (OR) along with 95% confidence intervals (CI). The statistically significant variables which influence SRH in univariate analyses were subsequently incorporated into the final model of multivariate logistic regression. The model was adjusted by the prevalence of chronic diseases by means of the backward selection method. The level of confidence applied was p<0.05. The Hosmer-Lemenshow test was used for testing the goodness of fit. The statistical analysis was performed using the SPSS v. 19.0 software.

## Results

**Fig. 1:**
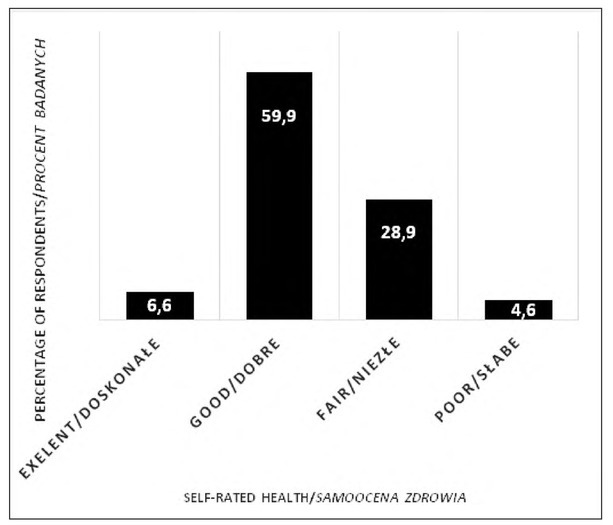
Self-rated health of 15-year-old girls. Ryc. 1. Samoocena zdrowia 15-letnich dziewcząt.

Two thirds of the girls assessed their health as “excellent” and “good” (6.6% and 59.9%, respectively) and 4.6% of the respondents made the extreme, negative assessment (Fig. 1).

Table I presents the frequency of high SRH scores among girls and the results of univariate logistic regression analysis depending on selected health behaviours. Compared to the girls with adverse health behaviours, those characterised by health-promoting behaviours more often assessed their health as “excellent” and “good” (Tab. I). Regular physical activity, consumption of breakfast, fruits and vegetables every day, as well as frequent family meals were found to increase the chance of a high SRH score. Moreover, enough sleep, avoidance of alcohol and shorter screen time were also proved to contribute to better SRH scores. Out of the 13 health behaviours, only two: nonsmoking and non-use of marijuana, were not significantly associated with high SRH scores among girls.

In the univariate model, the highest probability of high SRH scores was found in girls regularly participating in PE classes and then by those having breakfast every day, characterised by MVPA 60 min/7days/week and VPA >2-3 days/week.

Afterwards, a multivariate logistic regression model was estimated to identify the factors having the greatest impact on high SRH scores in the girls examined (Tab. II). In the model that was created and adjusted for chronic diseases or disability, the most important factors proved to be regular participation in PE classes, which was almost as strong a predictor as the absence of a chronic disease or disability. The following factors were other important predictors of high SRH scores: consumption of fruits at least once a day, breakfast consumption every day, VPA >2-3 times a week and sleep time of at least 8 hours a day. Family meals were on the borderline of significance (p=0.079), while the impact of drinking alcohol was not statistically insignificant (p=0.098).

The Hosmer-Lemenshow test showed a good fit of data to the final regression model (p=0.815). Such variables as MVPA, consumption of vegetables, using a tablet and a smartphone did not enter the final model.

## Discussion

Self-rated health is a particularly important health status indicator for teenage girls. Gender significantly differentiates self-rated health and is worse for girls at puberty [3, 8, 13,14]. In this study we discuss SRH of a representative group of 15-year-old girls, in the context of behavioural factors, both beneficial and risk behaviours.

**Table I:** Frequency of high self-rated health in girls and results of univariate analysis by selected health behaviours. Tabela I. Częstość występowania wysokiej samooceny zdrowia u dziewcząt oraz wyniki analizy jednowymiarowej w zależności od wybranych zachowań zdrowotnych.

Health behaviour *Zachowania zdrowotne*	Categories *Kategorie*	High SRH *Wysoka SRH (%)*	P*	OR	95% Cl	p**
**Nutritional behaviows*/Zachowania związane z odżywianiem***						
Breakfast consumption on school days *Jedzenie śniadań w dni szkolne*	Every *day/codziennie* <5 days (^)/<5 *dni*	71.8 60.7	0.000	1.65 1.00	1.29-2.10	0.000
Consumption of fruits *Jedzenie owoców*	At least once a day *Przynajmniej raz dziennie* Less often than once a day (^) *Rzadziej niż raz dziennie*	72.3 63.3	0.002	1.52 1.00	1.17-1.97	0.002
Consumption of vegetables *Jedzenie warzyw*	At least once a day *Przynajmniej raz dziennie* Less often than once a day (^) *Rzadziej niż raz dziennie*	70.2 64.3	0.040	1.31 1.00	1.01-1.70	0.040
Family meals *Posiłki spożywane wspólnie z rodziną*	Every day or on most days of the week *Less often (^)*	70.8 61.2	0.001	1.54 1.00	1.20-1.96	0.001
**Behaviours related to physical activity*/Zachowaniazwiązane z aktywnością fizyczną***						
MVPA *Umiarkowana aktywność fizyczna*	=7days/week *codziennie* <7days/week (^) *mniej niż 7dni w tygodniu*	75.7 65.2	0.011	1.66 1.00	1.12-2.45	0.012
VPA *Intensywna aktywność fizyczna*	> 2-3 days/week *2-3 dni w tygodniu lub więcej <2* days/week (^) *mniej niż 2 dni w tygodniu*	70.1 59.8	0.000	1.50 1.00	1.23-2.03	0.000
Participation in physical education classes*Udział w lekcjach wf*	Regula*r/Regularny* Irregular (^)/*Nieregularny*	70.4 56.6	0.000	1.82 1.00	1.40-2.38	0.000
**Sedentary behaviours/*Zachowonio sedentarne***						
Watching TV and DVD on school days*Oglądanie TV i DVD w dni szkolne*	<2 hour/day *2 godziny dziennie lub mniej >2* hour/day (^) *więcej niż 2 godziny dziennie*	68.9 61.5	0.011	1.38 1.00	1.08-1.79	0.011
Using social media on school days *Korzystanie z mediów* *społecznościowych w dni szkolne*	*<2* hour/day 2 *godziny dziennie lub mniej* *>2* hour/day (^) *więcej niż 2 godziny dziennie*	71.8 62.9	0.002	1.50 1.00	1.17-1.93	0.002
**Risk behaviours/*Zachowania ryzykowne***						
Tobacco smoking *Palenie tytoniu*	No/*Nie* Yes (^)/*Tak*	66.9 62.9	0.450	1.19 1.00	0.76-1.86	0.450
Drinking alcohol throughout lifetime *Picie alkoholu w całym życiu*	No/*Nie* Yes (^)/*Tak*	69.3 61.9	0.010	1.39 1.00	1.08-1.78	0.010
Using drugs *Używanie narkotyków*	No/*Nie* Yes (^)/Tok	66.9 55.3	0.135	1.64 1.00	0.85-3.14	0.138
**Sleep/Sen**						
Amount of sleepon school days *Ilość snu w dni szkolne*	>8 hour/day *8 lub więcej godzin na dobę* <8 hour/day (^) *mniej niż 8 godzin na dobę*	70.5 62.8	0.006	1.42 1.00	1.10-1.82	0.006

^ Reference grou*p/Grupa odniesienia* *Value of Pearson's Chi-squared test/*Wortość testu Chi-kwadrat Pearsona* **Based on the results of logistic regression/*Na podstawie wyników regresji logistycznej*

**Table II:** Multivariate logistic regression model for high SRH of 15-year-old girls. Tabela II. Model wielowymiarowej regresji logistycznej dla wysokiej samooceny zdrowia 15-letnich dziewcząt.

Explanatory variable *Zmienna objaśniająca*	B	Wald	P	OR	95% confidence interval for OR *95% przedział ufności dla OR*	
					Lower limit *Dolna granica*	Upper limit *Górna granica*
Chronic disease *Choroba przewlekła* *No/Nie* Yes(^)*/Tak*	-0.521	9.252	0.002	0.59 1.00	0.43	0.84
Breakfast consumption on school days *Jedzenie śniadań w dni szkolne* Every day/*Codziennie* Irregularly (^)/*Nieregularnie*	0.337	6.232	0.013	1.40 1.00	1.08	1.83
Family meals *Spożywanie wspólnych posiłków z rodziną* *Often/Często* Seldom (^)*/Rzadko*	0.238	3.089	0.079	1.27 1.00	0.97	1.65
Consumption of fruit during the week *Jedzenie owoców* At least once a *day/Przynajmniej raz dziennie* Less often ^*)/Rzadziej*	0.415	8.220	0.004	1.51 1.00	1.14	2.01
Participation in PE classes *Udział w lekcji wf* Regular/*Regularny* Irregular (^) */Nieregularny*	0.449	9.552	0.002	1.57 1.00	1.18	2.08
VPA *Intensywna aktywność fizyczna* > 2-3 days/week/2-3 *dni w tygodniu lub więcej* *<2* days/week (^)/*mniej niż 2 dni w tygodniu*	0.329	5.598	0.018	1.39 1.00	1.06	1.78
Drinking alcohol throughout lifetime *Picie alkoholu w całym życiu* *tiever/Nigdy* Varied frequency (^)/Z *różną częstością*	0.229	2.744	0.098	1.26 1.00	0.96	1.65
Amount of sleep on school days *Ilość snu w dni szkolne* At least 8 hours/*Przynajmniej 8 godzin* Less than 8 hours (^)*/Mniej niż 8 godzin*	0.269	3.964	0.046	1.31 1.00	1.01	1.71
Constant/*Stała*	-0.437	5.214	0.022	0.646		

*Reference group/Grupo *odniesienia*

Two thirds of the 15-year-olds analysed gave high scores to their health assessment (excellent and good). A relatively large percentage, almost 30%, thought that their their health was fair (in international studies “fair” achieved the average value of approximately 12% in 2014), which may mean that for Polish teenage girls this category is associated rather positively or at least neutrally, but does not mean “good” health [15]. The fact has also been observed by other researchers who argue that SRH, as a reflection of the subjective physical, mental and social state of individuals does not have to be understood in the same way by individuals in various groups and in various countries [16]. Despite those limitations, the indicator is extensively used in research.

Our study found an association between high SRH scores and health behaviours. The most important factor among the behavioural factors checked in the subsequent step of the analysis, i.e. multivariate logistic regression adjusted for the occurrence of a chronic disease or disability, proved to be the impact of regular participation in PE classes. This was almost as strong a predictor as the absence of a chronic disease or disability and constitutes very important information due to the fact that in general Polish adolescents tend to avoid PE classes.

The study carried out in the 2013/2014 school year on a representative sample of over 3000 school children aged 10-17 years showed that for about 20-40% students, the number of PE classes they attended had been considerably lower than those planned in the core curriculum. The participation of students in PE classes decreased with age, was lower for girls than for boys, and for older students than for younger ones. Girls more often than boys did not participate at all or hardly ever participated in such classes (4.3% vs. 2.7%). Almost half of the students (42.9%) excused themselves from the classes with a PE teacher at least once a school year. Girls excused themselves more often (49.7%) than boys (35.7%). This may be one of the reasons for their lower level of physical activity than boys [17].

Regular attendance of mandatory PE classes entails the participation of young people in organised sport activities and often vigorous physical outdoor activities. These often provide a competitive offer to spending leisure time in front of a screen. Sedentary activities, including screen time, were not included in the final regression model in this study on teenage girls, but they play an important role in predicting life satisfaction and SRH, as emphasized by numerous authors worldwide [18, 19, 20]. The present study showed that a physically active lifestyle is related to better SRH scores and well-being. Young people who do sports have a better quality of life than those who remain sedentary [3,21]. The authors emphasize that the association between physical activity and SRH is more visible in boys than in girls and pointed out that girls need to undertake more days of MVPA to obtain similar health benefits. Our study provides another argument in support of this claim, since VPA in teenage girls proved to be one of the most powerful predictors of high SRH scores.

Some authors believe that the frequency of breakfast consumption in adolescents is the most characteristic feature of their nutritional behaviour and lifestyle. A decrease in breakfast consumption is recorded mainly during puberty. It has been demonstrated that meal patterns, such as skipping breakfast, are associated with a group of lifestyle factors and dietary choices which are less beneficial for health and in consequence lead to reduced consumption of nutrients, abdominal obesity and the risk of the metabolic syndrome [22,23].

Osera et al. showed that morning routines are very important factors influencing the SRH of secondary school students, which may have an impact on their future overall health status. Both boys and girls with good “sleeping habits” (i.e. those who reported that they go to bed early and wake up early) may have enough time to have breakfast every morning [24]. The results of our studies seem to confirm this finding in the analysed girls, since everyday breakfast consumption and sleeping for at least 8 hours were important predictors of their high SRH score. Zhang et al. confirmed that various sleep problems, such as interrupted or insufficiently long sleep, correlate with poor SRH scores [25], while Foti et al. found that night sleep of over 8 hours is one of the main predictors of a very good SRH score in females [6].

In the studies mentioned, Foti at al. also referred to the association between SRH assessment and risk behaviours. They show that out of all the ethnic groups from the vocational school students in the USA that they analysed, an increased number of risk behaviours was associated with low SRH scores. Although the indicators of excellent SRH scores among teenagers were not analysed, the authors suggest that factors other than risk behaviours contribute to the adolescents’ perception of health as excellent. This theory seems to be confirmed by our study where in the final step of the analyses no association was found between risk behaviours and high SRH scores.

Research on SRH among adolescents focuses mainly on negative self-assessment and related factors. From the perspective of health promotion, it seems that a positive approach to health and searching for health determinants, i.e. factors conducive to health, is more beneficial than looking for reasons for poor SRH scores [26].

## Conclusions

The results showed that regular participation in PE classes, VPA, eating breakfast and fruits every day and sleeping at least 8 hours a day are powerful predictors of high SRH scores among 5-year-old girls. Extensive promotion of such behaviours among adolescents at school and perhaps primarily in the social media, may contribute to maintaining good health and an optimal level of functioning among adolescents now and in the future.
